# CpG Sites Associated with Cigarette Smoking: Analysis of Epigenome-Wide Data from the Sister Study

**DOI:** 10.1289/ehp.1307480

**Published:** 2014-04-04

**Authors:** Sophia Harlid, Zongli Xu, Vijayalakshmi Panduri, Dale P. Sandler, Jack A. Taylor

**Affiliations:** 1 Laboratory of Molecular Carcinogenesis, and; 2 Epidemiology Branch, National Institute of Environmental Health Sciences, National Institutes of Health, Department of Health and Human Services, Research Triangle Park, North Carolina, USA; * These authors contributed equally to this work.

## Abstract

Background: Smoking increases the risk of many diseases, and it is also linked to blood DNA methylation changes that may be important in disease etiology.

Objectives: We sought to identify novel CpG sites associated with cigarette smoking.

Methods: We used two epigenome-wide data sets from the Sister Study to identify and confirm CpG sites associated with smoking. One included 908 women with methylation measurements at 27,578 CpG sites using the HumanMethylation27 BeadChip; the other included 200 women with methylation measurements for 473,844 CpG sites using the HumanMethylation450 BeadChip. Significant CpGs from the second data set that were not included in the 27K assay were validated by pyrosequencing in a subset of 476 samples from the first data set.

Results: Our study successfully confirmed smoking associations for 9 previously established CpGs and identified 2 potentially novel CpGs: cg26764244 in *GNG12* (*p* = 9.0 × 10^–10^) and cg22335340 in *PTPN6* (*p* = 2.9 × 10^–05^). We also found strong evidence of an association between smoking status and cg02657160 in *CPOX* (*p* = 7.3 × 10*^–^*^7^), which has not been previously reported. All 12 CpGs were undermethylated in current smokers and showed an increasing percentage of methylation in former and never-smokers.

Conclusions: We identified 2 potentially novel smoking related CpG sites, and provided independent replication of 10 previously reported CpGs sites related to smoking, one of which is situated in the gene *CPOX*. The corresponding enzyme is involved in heme biosynthesis, and smoking is known to increase heme production. Our study extends the evidence base for smoking-related changes in DNA methylation.

Citation: Harlid S, Xu Z, Panduri V, Sandler DP, Taylor JA. 2014. CpG sites associated with cigarette smoking: analysis of epigenome-wide data from the Sister Study. Environ Health Perspect 122:673–678; http://dx.doi.org/10.1289/ehp.1307480

## Introduction

Cigarette smoking is a known risk factor and contributor to adverse health outcomes such as cancer. The health effects associated with smoking are mediated through a variety of mechanisms, including direct DNA damage, increased inflammation, and others (Hecht 2012; Mathers and Loncar 2006; Takahashi et al. 2010; Witschi et al. 1997). One of the ways by which smoking contributes to disease may be through epigenetic changes such as DNA methylation (Portela and Esteller 2010).

Epigenome-wide association studies (EWAS) of DNA methylation in blood have recently identified a number of CpG loci associated with adult smoking (Breitling et al. 2011; Shenker et al. 2013a; Sun et al. 2013; Wan et al. 2012; Zeilinger et al. 2013). The first EWAS to investigate the association between smoking and DNA methylation made use of the 27K array (Infinium HumanMethylation27 BeadChip; Illumina, San Diego, CA), and identified a differentially methylated CpG site in *F2RL3* [coagulation factor II (thrombin) receptor-like 3] that showed decreased methylation in smokers (Breitling et al. 2011). More recent studies have made use of the newer 450K methylation array (Infinium HumanMethylation450 BeadChip; Illumina) (Shenker et al. 2013a; Sun et al. 2013; Zeilinger et al. 2013) to uncover new associations between smoking and DNA methylation and they have all confirmed the initial results in *F2RL3*. Another gene, *AHRR* (aryl hydrocarbon receptor repressor), has consistently been a top finding in these studies.

One large EWAS investigated the association between DNA methylation in cord blood and maternal smoking (Joubert et al. 2012). Joubert et al. (2012) used the 450K methylation bead chip (Illumina) and their top hit was also *AHRR*. There are also several reports of smoking-associated changes in global DNA methylation as well as changes in candidate genes (Breitling et al. 2012; Hillemacher et al. 2008; Kiseljak-Vassiliades and Xing 2011; Philibert et al. 2010; Smith et al. 2007; Tekpli et al. 2012; Wolff et al. 2008; Zhang et al. 2011).

Two of the five EWAS (Breitling et al. 2011; Wan et al. 2012) made use of the 27K methylation array, which assesses methylation at 27,578 CpG sites; however, this array lacks coverage at many potentially relevant sites. Three studies made use of the newer Illumina Infinium HumanMethylation450 BeadChip, which measures methylation at 473,844 CpG sites, and only one of these had a substantial sample size (Zeilinger et al. 2013).

We examined smoking and DNA methylation in a nationwide sample of volunteer women to confirm previously reported results and identify new sites associated with smoking. We analyzed two sets of methylation array data from the Sister Study—a prospective cohort study focused on environmental and familial risk factors for breast cancer and other diseases in women with a sister diagnosed with the disease.

## Methods

*Study design*. All study subjects were participants of the National Institute of Environmental Health Sciences (NIEHS) Sister Study, a nationwide prospective cohort study designed to examine genetic and environmental determinants of breast cancer. To be eligible for the Sister Study, women could not have had breast cancer themselves but must have had a biological sister with breast cancer. Detailed information can be found online at the Sister Study website (NIEHS 2014). At baseline all participants provided a blood sample and completed an extensive questionnaire on smoking history, including current smoking status, years since quitting, amount, and duration (NIEHS 2014). Informed consent was obtained from all participants before participation. The study was approved by the institutional review boards of the NIEHS, National Institutes of Health, and the Copernicus Group (http://www.cgirb.com/irb-services/).

Our first sample included 908 non-Hispanic white women between 35 and 75 years of age. Blood methylation status in these women had been determined previously using the HumanMethylation27 BeadChip as part of a case–cohort study of methylation and breast cancer. The original study (Xu et al. 2013) utilized a nested case–cohort study design and included all incident breast cancer cases diagnosed between blood draw and May 2008 (*n* = 329). The control group included a random sample of women drawn from the 29,026 participants enrolled in the Sister Study by June 2007 (*n* = 709). Some samples were later excluded due to lack of DNA or poor quality measurements. For the present study we included only non-Hispanic white women in order to account for factors affected by race. Our final sample therefore included 296 women who had been diagnosed with cancer within 46 months of blood draw and a random sample of 612 women who had remained cancer free for up to 55 months of follow-up (Xu et al. 2013). Here we refer to this group as the 27K data set.

Our second group of Sister Study participants included 200 women whose blood methylation was measured as part of an unpublished pilot study examining potential prenatal exposure to diethylstilbestrol (DES). In this study, all women were non-Hispanic white between the ages of 41 and 59 years. All women in this sample set were originally selected from a subset of 1,802 women from a special substudy validating self-reported exposures with the participant’s mothers. A total of 100 exposed and 100 unexposed non-Hispanic white women were selected (unexposed women were frequency matched on age). Samples from all 200 women were successfully assayed using the HumanMethylation450 BeadChip. We refer to this group as the 450K data set.

*Infinium methylation assays*. The HumanMethylation27 BeadChip provides DNA methylation data at single CpG–site resolution for 27,578 different CpG sites covering promoter regions for > 14,000 Human RefSeq genes across 23 chromosomes. The HumanMethylation450 BeadChip provides information on 485,577 CpG sites with coverage of 99% of RefSeq genes and an average of 17 CpG sites per gene including sites in the promoter, 5´UTR, first exon, gene body, and 3´UTR. A total of 25,978 CpG sites are shared between the HumanMethylation27 and HumanMethylation450 BeadChip arrays.

DNA from both sets of women was extracted from frozen whole blood samples as previously described (Xu et al. 2013). Extracted DNA was quantified using Quant-iT^TM^ PicoGreen dsDNA reagent (Invitrogen, Carlsbad, CA) and stored at –20°C. One microgram of DNA was bisulfite converted using the EZ-DNA Methylation kit (Zymo Research, Irvine, CA) following the manufacturer’s protocol. DNA was hybridized to the HumanMethylation27 or HumanMethylation450 BeadChip arrays following the manufacturer’s protocol and then scanned with an iScan microarray scanner (Illumina). Data were analyzed using Illumina GenomeStudio® software (version 2011.1).

At each CpG site on the array, methylation status was determined based on intensity measures of two probes corresponding to unmethylated (U) or methylated (M) CpGs. Dye bias between U and M for type II probes in the HumanMethylation450 BeadChip was corrected using the normalizeMethyLumiSet method in R package “methylumi_2.4.0” (http://www.r-project.org/foundation/). Before association analysis, the intensity values were separately robust multichip average–background corrected (Irizarry et al. 2003) and quantile normalized across arrays. The methylation level (beta value) of a specific CpG site was calculated as the ratio of normalized fluorescent intensities between methylated and unmethylated alleles β *=* M*/*(M *+* U *+* 100). To avoid SNP (single-nucleotide polymorphism) effects on methylation measures, we excluded CpG probes with SNPs present at target sites (428 CpGs from the HumanMethylation27 BeadChip and 20,869 CpGs from the HumanMethylation450 BeadChip). In both data sets a nonspecific filtering step was applied to filter out the 20% CpGs with the smallest interquartile range (IQR) of methylation values before association analysis. In the 27K data set we tested 21,659 probes and in the 450K data set we tested 369,120 probes.

*Pyrosequencing*. Pyrosequencing assays were developed for two CpGs [cg02657160 in *CPOX* (coproporphyrinogen oxidase) and cg15999356 in *YAP1* (Yes-associated protein 1)] that showed potentially novel associations with smoking in the 450K data set. Neither of the CpGs was present on the HumanMethylation27 BeadChip, and thus the women who were members of the 27K data set had not been evaluated at these two sites. For pyrosequencing analysis we selected 68 current smokers from the 27K data set, and for each current smoker we selected three former smokers and three never-smokers matched for age at blood draw and bisulfite conversion batch (Table 1).

**Table 1:** Sample characteristics for participants in the data sets (n or mean ± SD).

Characteristic	Never-smoker	Former smoker	Current smoker
27K data set
Participants	496	344	68
Age (years)	55.13 ± 9.09	57.07 ± 8.83	53.18 ± 9.63
Pack-years	—	13.24 ± 13.93	25.17 ± 13.58
Years of smoking	—	15.13 ± 10.64	32.64 ± 11.46
Age started smoking (years)	—	17.92 ± 3.96	17.25 ± 3.57
Years since quitting	—	22.03 ± 10.71	—
Breast cancer cases/non-cases	165/331	111/233	20/48
450K data set
Participants	118	70	12
Age (years)	50.31 ± 4.80	51.16 ± 5.04	50.50 ± 3.55
Pack-years		9.92 ± 11.46	24.45 ± 15.34
Years of smoking	—	11.70 ± 9.06	29.63 ± 6.84
Age started smoking (years)	—	17.41 ± 3.70	16.25 ± 2.56
Years since quitting	—	20.94 ± 9.35	—
DES exposed/nonexposed	60/58	34/36	6/6
Pyrosequencing
Participants	204	204	68
Age (years)	53.14 ± 9.50	56.11 ± 8.91	53.18 ± 9.63
Pack-years		18.37 ± 14.71	25.17 ± 13.58
Years of smoking	—	20.44 ± 10.06	32.64 ± 11.46
Age started smoking (years)	—	17.03 ± 3.22	17.25 ± 3.57
Years since quitting	—	16.79 ± 8.42	—

Pyrosequencing primers for cg02657160 (*CPOX*) and cg15999356 (*YAP1*) (see Supplemental Material, Table S1) were designed using Pyromark Assay Design version 2.0.2.15 (Qiagen, Valencia, CA). Reaction mixtures (25 μL) containing 100ng of bisulfite converted DNA, 5 pmol of each primer (forward and reverse) polymerase chain reaction (PCR) buffer (Invitrogen), 3 mM MgCl_2_, 1 mM dNTP, and 0.8 units of taq polymerase (Invitrogen), were heated to 95°C for 15 min, followed by 45 PCR cycles (95°C for 20 sec, 55°C for 20 sec and 72°C for 20 sec) with a final extension at 72°C for 5 min. After PCR, the biotin-labeled PCR product was hybridized to streptavidin-coated sepharose beads (GE Healthcare, Madison, WI) and denatured in 0.2 M sodium hydroxide to provide a single-stranded sequencing template. Pyrosequencing primers (0.3 μmol/L) were annealed to the single-stranded template and the pyrosequencing was carried out using PyroMark Q96 MD System (Qiagen) according to the manufacturer’s instructions. The percentage methylation was quantified using the Pyro Q-CpG Software (Qiagen).

*Statistical analysis*. A multiple linear regression model was employed to test the association between smoking status [never, former, and current smoker (see Supplemental Material, “Smoking Variable,” p. 2, for a complete description of this variable) and methylation (β value) at each CpG site, adjusting for age at blood draw. In the 27K data set we also adjusted for case–control status and in the 450K data set we adjusted for DES exposure. A surrogate variable analysis (Leek and Storey 2007) was carried out in the methylation β value matrix to derive an additional set of variables. We then adjusted for these variables in the multiple linear regression model, thereby accounting for known and unknown confounders such as experimental batch effect. To examine whether additional variables such as passive smoking were influencing our results, we analyzed the correlation between our primary smoking variables and passive smoking, maternal smoking, parity, and menopausal status as well by adjusting for these variables. All analyses were repeated only in the breast cancer-free women (27K data set) but not in the DES-unexposed women from the 450K data set because this would have produced a highly underpowered analysis due to the small sample size.

To minimize the effect of outliers, for each CpG probe we excluded approximately 0.8% of the β values that were > 3 SDs from the mean. To correct for multiple testing, we estimated the false discovery rate (FDR) using the *q*-value framework (Storey and Tibshirani 2003). Effects of years of smoking, pack-year, and time since quitting on methylation changes were tested using multiple linear regression adjusting for age at blood draw. When analyzing the pyrosequencing data, we employed a two-way analysis of variance (ANOVA) method to model the 1:3:3 matched study design and tested whether the methylation level in current smokers or former smokers differed from the methylation level never-smokers.

## Results

Characteristics of study participants according to smoking status are summarized in Table 1. Former smokers were slightly older than never-smokers and current smokers. Current smokers had, on average, longer smoking history than former smokers. The smoking characteristics of women in the 27K data set did not differ between breast cancer cases and non-cases; we therefore included both groups in the analysis with adjustment for case status. This was also true for the 450K data set where there was no difference between exposed and unexposed women. The 27K data set included 496 (54.6%) never-smokers, 344 (37.9%) former smokers, and 68 (7.5%) current smokers, and the 450K data set included 118 (59%) never-smokers, 70 (35%) former smokers, and 12 (6%) current smokers. The subset of the 27K data set that was used for pyrosequencing included 204 (42.8%) never-smokers, 204 (42.8%) former smokers, and 68 (14.2%) current smokers (Table 1).

**Figure 1:**
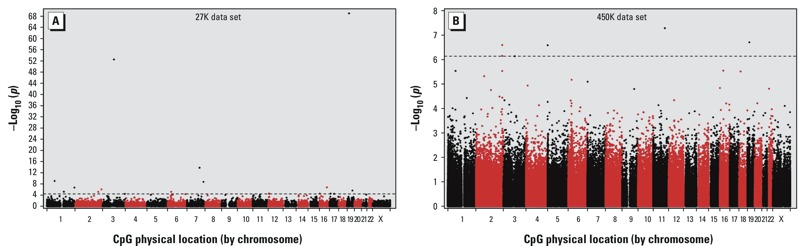
Manhattan plots of smoking epigenome-wide association p-values for the (*A*) HumanMethylation27 BeadChip or the (*B*) HumanMethylation450 BeadChip assay. Horizontal dashed lines indicate the FDR threshold of 0.05.

**Figure 2:**
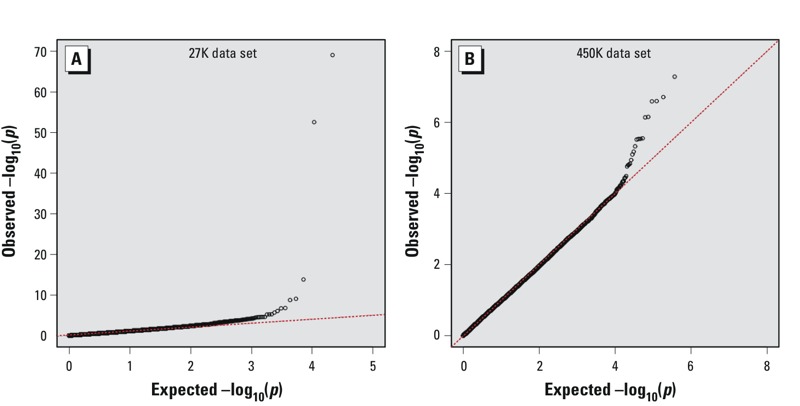
Q–Q plots for smoking epigenome-wide association *p*-values for the 27K data set (*A*) and the 450K data set (*B*). Values shown are –log_10_ transformed expected *p*-values and –log_10_ transformed observed *p*-values.

Using the 27K data set as a discovery set, there were 18 CpGs associated with smoking status at false discovery rate of *q* < 0.05 (Figure 1A, Figure 2A; see also Supplemental Material, Table S2). Seventeen of the 18 identified CpGs were available for replication analysis in the 450K data set (cg13185177 was not present on the 450K array and thus could not be further examined). Eight of the 17 CpGs were confirmed in the 450K data set at an *a priori p*-value threshold of 0.05 (Table 2). Although the percent methylation estimates for each CpG varied somewhat between the two array platforms, differences between never, former, and current smokers for each of the eight CpGs were similar between the two array platforms (Table 2). Smoking associations had been reported previously for six of the eight CpGs, whereas associations for the remaining two {cg22335340 in *PTPN6* [protein tyrosine phosphatase, non-receptor type 6] and cg26764244 in *GNG12* [guanine nucleotide binding protein (G protein)]} had not been previously reported (Table 2).

**Table 2:** Differentially methylated CpG sites by smoking status at an FDR threshold of 0.05 (27K data set) and validated with a *p* < 0.05 (450K data set).

CpG	Gene	27K data set					450K data set				Reference^*a*^
		β-Value never	β-Value former	β-Value current	*p*-Value	FDR	β-Value never	β-Value former	β-Value current	*p*-Value	
cg03636183	*F2RL3*	0.85	0.83	0.78	9.3 × 10^–70^	1.9 × 10^–65^	0.79	0.77	0.7	2.0 × 10^–07^	Breitling et al. 2011; Shenker et al. 2013a; Sun et al. 2013; Wan et al. 2012; Zeilinger et al. 2013
cg19859270	*GPR15*	0.84	0.83	0.81	2.9 × 10^–53^	3.0 × 10^–49^	0.95	0.94	0.93	3.8 × 10^–04^	Breitling et al. 2011; Sun et al. 2013; Wan et al. 2012; Zeilinger et al. 2013
cg09837977	*LRRN3*	0.76	0.75	0.74	1.7 × 10^–14^	1.2 × 10^–10^	0.88	0.87	0.83	5.0 × 10^–04^	Sun et al. 2013; Wan et al. 2012
cg26764244	*GNG12*	0.12	0.11	0.098	9.0 × 10^–10^	4.6 × 10^–06^	0.26	0.24	0.22	0.006	NA
cg16254309	*CNTNAP2*	0.063	0.06	0.053	1.9 × 10^–09^	7.7 × 10^–06^	0.064	0.06	0.039	0.01	Joubert et al. 2012; Shenker et al. 2013a; Sun et al. 2013; Wan et al. 2012; Zeilinger et al. 2013
cg13500388	*CBFB*	0.43	0.42	0.41	1.8 × 10^–07^	6.0 × 10^–04^	0.55	0.54	0.51	0.02	Sun et al. 2013
cg11314684	*AKT3*	0.27	0.26	0.25	2.1 × 10^–07^	6.2 × 10^–04^	0.35	0.34	0.31	9.3 × 10^–04^	Sun et al. 2013
cg22335340	*PTPN6*	0.65	0.65	0.64	2.9 × 10^–05^	4.0 × 10^–02^	0.78	0.77	0.75	0.01	NA
Abbreviations: *CNTNAP2*, contactin associated protein-like 2; *GPR15*, G protein-coupled receptor 15; *LRRN3*, leucine rich repeat neuronal 3; NA, not applicable. ^***a***^Previous publications where the CpG has been reported.											

When analyzing the correlation between the primary smoking variables and other potential confounders, secondhand smoking exposure was highly correlated with the primary smoking variables, whereas none of the other potential confounders were correlated (data not shown). After adjustment, all except for one of the probes (cg22335340 in PTPN6) reported in Table 2 were still statistically significant at the FDR threshold of 0.05; there was no statistically significant association with passive smoking (see Supplemental Material, Table S3). We repeated the analysis in the 612 breast cancer–free women from the 27K data set (see Supplemental Material, Table S4). When repeating the analysis in non-cases, six of the probes remained significant and two probes [cg13500388 in CBFB (core-binding factor, beta) and cg11314684 in AKT3 (v-akt murine thymoma viral oncogene homolog 3)] failed to reach significance compared with when we used the data set containing both cases and non-cases. This could possibly be due to small sample size.

We also used the 450K data set as a second discovery set to look for new CpGs associated with smoking. Because of the smaller sample size in this data set, we combined former smokers and current smokers into a single group for smoking association analysis. After excluding the 17 CpGs already examined as part of the replication study, 5 CpGs were significantly associated with smoking status at a study-wide FDR threshold of 0.05 (Figures 1B, 2B, Table 3). Three of the five CpGs had previously been reported to be associated with smoking, whereas two, cg15999356 in *YAP1* and cg02657160 in *CPOX,* had not been previously reported at the time our experiments were designed.

**Table 3:** Cigarette smoking–related CpG sites identified in the 450K data set and confirmation results by pyrosequencing experiment.

CpG	Gene	450K data set					Pyrosequencing				Reference^*a*^
		β-Value never	β-Value former	β-Value current	*p*-Value	FDR	β-Value never	β-Value former	β-Value current	*p*-Value	
cg02657160	*CPOX*	0.87	0.86	0.84	7.3 × 10^–07^	4.5 × 10^–02^	0.88	0.87	0.86	1.1 × 10^–11^	Zeilinger et al. 2013
cg15999356	*YAP1*	0.79	0.74	0.76	5.2 × 10^–08^	1.9 × 10^–02^	0.64	0.63	0.63	0.23	NA
cg05575921	*AHRR*	0.85	0.83	0.75	2.6 × 10^–07^	2.4 × 10^–02^	NA	NA	NA	NA	Joubert et al. 2012; Shenker et al. 2013a; Sun et al. 2013; Zeilinger et al. 2013
cg06644428	2q37.1^*b*^	0.1	0.081	0.043	2.6 × 10^–07^	2.4 × 10^–02^	NA	NA	NA	NA	Shenker et al. 2013a; Sun et al. 2013; Zeilinger et al. 2013
cg05951221	2q37.1^*b*^	0.56	0.53	0.41	7.1 × 10^–07^	4.5 × 10^–02^	NA	NA	NA	NA	Shenker et al. 2013a; Sun et al. 2013; Zeilinger et al. 2013
Abbreviations: 2q37.1, intergenic CpG island on chromosome 2q37; NA, not applicable. ^***a***^Previous publications where the CpG has been reported. ^***b***^Refers to a chromosome location.											

As an independent replication, we used pyrosequencing to examine the *YAP1* and *CPOX* CpGs in 476 women who originally participated in the 27K study (which did not include these two sites on the 27K array). Although cg15999356 in *YAP1* showed little evidence of association with smoking, (*p* = 0.23), there was strong evidence of association for cg02657160 in *CPOX* (Table 3; see also Supplemental Material, Figure S1, two-way ANOVA, *p* = 1.1 × 10^–11^).

Including all CpGs discovered and replicated in the 27K data set (8 CpGs, Table 2), and the 450K data set (4 CpGs, Table 3), our study could confirm smoking associations for 10 established and 2 potentially novel CpGs. There was a consistent ordering of methylation values across all but 2 of the 12 CpGs, with highest values in never-smokers, intermediate values in former smokers, and lowest values in current smokers; all 12 CpGs exhibited the lowest methylation values in current smokers (Tables 2 and 3).

Among former smokers, methylation values for all 12 CpG sites showed decreasing methylation with increasing number of years smoked and increasing pack-years, although these findings were not statistically significant at all sites (see Supplemental Material, Tables S5, S6). Similarly, among former smokers, methylation levels consistently increased with time since quitting smoking, although again these results were not statistically significant at all sites (see Supplemental Material, Tables S5, S6). These findings were somewhat less consistent (both for direction and statistical significance) among current smokers, perhaps reflecting the smaller sample size of this group. Using multiple linear regression, we found a significant interaction between the number of years smoked and time since quitting on methylation for three CpG sites: cg03636183 at *F2RL3* (*p* = 2.73 × 10^–4^), cg19859270 at *GPR15* (G protein-coupled receptor 15) (*p* = 1.98 × 10^–2^), and cg22335340 at *PTPN6* (*p* = 2.91 × 10^–3^). These three CpGs were also inversely associated with smoking pack-years, but the association disappeared after adjusting for number of years smoked (data not shown).

## Discussion

We performed an EWAS to investigate the effect of cigarette smoking on DNA methylation using previously collected data from the Sister Study. We confirmed (at a threshold of *p* < 0.05) smoking-associated CpGs for nine CpGs (eight discovered in the 27K data set and one discovered in the 450K data set). Two of these nine CpGs remain unreported (cg22335340 in *PTPN6* and cg26764244 in *GNG12*). One CpG (cg02657160 in *CPOX*), was recently reported as a supplementary result (Zeilinger et al. 2013), but aside from this its association with smoking has not been described. We have summarized reported smoking-associated CpGs and our association *p*-values in Supplemental Material, Table S7.

The most noteworthy finding of our study is the fact that we were able to successfully replicate a large number of previously identified CpG sites associated with cigarette-smoking exposure. This further strengthens the validity of their association with smoking and contributes information about the magnitude of effect that smoking has on DNA methylation levels in blood.

In line with most previous studies, we found that DNA methylation varies between current smokers and former smokers for most significant CpG sites. This may make it a useful biomarker of smoking status (Shenker et al. 2013b) and also indicates that these changes are stable for years and may continue to contribute to the increased risk of adverse outcomes associated with smoking.

**Figure 3:**
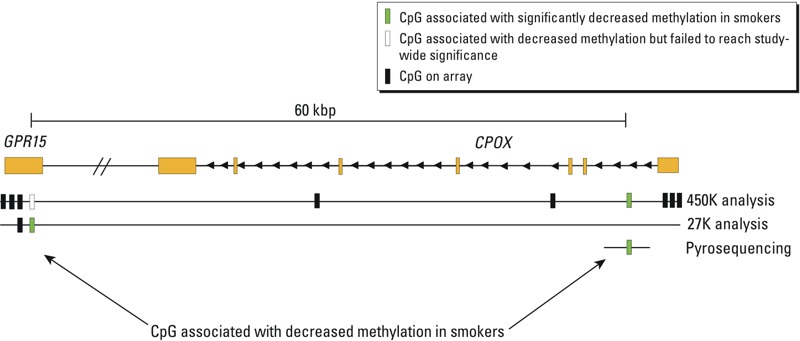
Depiction of the region surrounding cg02657160, which is located in the first intron of *CPOX*, 60 kbp from another CpG in *GPR15* that was identified both in the present study and by others (Wan et al. 2012). This suggests that a larger area may be affected by methylation changes.

The confirmation of cg19859270 in *CPOX* is particularly interesting because it is situated only 60 kbp upstream of another smoking-associated CpG in *GPR15* (cg19859270) that was also significant in our study and was previously identified by Wan et al. 2012 (Figure 3). *GPR15* codes for a G-protein–coupled receptor and functions as a co-receptor for human immunodeficiency virus (Blaak et al. 2005). Interestingly, gene expression of *GPR15* has been shown to increase in B cells from smokers compared with never-smokers (Pan et al. 2010). There are > 450 CpG sites that lay between the *CPOX* CpG and the *GRP15* CpG, but these sites are not included in either the 27K or 450K arrays and thus this region may be of interest for future study.

CPOX converts coprotoporphyrin III to protoporphyriogen IX, a reaction that constitutes the sixth step in heme biosynthesis (Layer et al. 2010). Heme is central in the synthesis of hemoglobin and is also important for the function of a large number of other proteins, including the cytochromes P450s (Correia et al. 2011). Rare inherited mutations in *CPOX* cause the autosomal dominant disease coproporphyria, which, like other porphyrias, is associated with low hemoglobin levels and anemia (Puy et al. 2010; Schmitt et al. 2005). Smoking is known to increase hemoglobin demands and can trigger coproporphyria attacks (Layer et al. 2010; Lip et al. 1991; Sassa 2006) it is also associated with both higher red blood cell counts and increased metabolism of cytochrome P450 enzymes (Schwartz and Weiss 1994; Van Tiel et al. 2002). A plausible connection between smoking and CPOX is that smoking exposure increases the demand for heme synthesis, which may result in increased expression of *CPOX*. The observed DNA methylation changes in *CPOX*, although small (≤ 2%), reflect a change in methylation, and presumably gene expression, in a small population of cells, although average gene expression across the cell population is unlikely to be altered.

We also observed smoking associations with DNA methylation at two potentially novel CpG sites: cg22335340 in *PTPN6* and cg26764244 in *GNG12*. The *PTPN6* CpG is located 500 bp upstream of the transcriptional start site. *PTPN6* codes for the protein SHP-1 (protein tyrosine phosphatase, non-receptor type 6), a protein tyrosine phosphatase that is a putative tumor suppressor gene (Wu et al. 2003). It is expressed in hematopoietic cells, and expression in lymphocytes from smokers is increased relative to levels among nonsmokers (Charlesworth et al. 2010), suggesting perhaps that the decreased DNA methylation we observe in smokers may be associated with increased transcriptional activity.

Cg26764244 in *GNG12* is also previously unreported in relation to smoking. However, two studies report an association between smoking and DNA methylation at another CpG in the same gene (cg25189904) (Shenker et al. 2013a; Zeilinger et al. 2013). Both cg26764244 and the previously reported cg25189904 are part of a close group of five CpGs (cg03140521, cg13184736, cg13399816, cg25189904 and cg26764244) situated in the south shore of a CpG island spanning the *GNG12* promoter (350 bp from the transcriptional start site). In our 450K data set, all five CpGs show decreased methylation in smokers and all have *p*-values < 0.05. The CpG reported by Shenker et al. (2013a) and Zeilinger et al. (2013), cg25189904, is not included on the 27K array; however, when we examine the methylation values in the 450K data set, it showed the largest methylation decrease and the smallest *p*-value of the CpGs in the five-CpG cluster (*p* = 2.94 × 10^–6^) suggesting that cg25189904 may be a CpG of interest.

We were also able to positively confirm (at genome-wide significance) eight CpGs in seven loci that have previously been described by others [*F2RL3, LRRN3, AKT3, CNTNAP2* (contactin associated protein-like 2), *CBFB, AHRR,* and 2q37.1 (an intergenic CpG island)] (Breitling et al. 2011; Shenker et al. 2013a; Sun et al. 2013; Wan et al. 2012). The top smoking-related finding in several studies is represented by cg05575921 in *AHRR*. This gene codes for the AhR repressor and is a component of the AhR signaling cascade, where it functions as a pathway inhibitor. The transcription factor AhR triggers expression of a diverse set of genes, some of which are involved in metabolism of endogenous substances including toxins from cigarette smoke (Haarmann-Stemmann et al. 2007; Kawajiri and Fujii-Kuriyama 2007; Swedenborg and Pongratz 2010). In addition, the relationship between smoking and the *AHRR* CpG has been identified in studies using DNA from both peripheral blood and pulmonary macrophages (Monick et al. 2012).

Similar to findings from previous reports (Shenker et al. 2013b; Wan et al. 2012; Zeilinger et al. 2013) we found evidence of an inverse association between pack-years and DNA methylation and a positive association between time since quitting smoking and DNA methylation. However, in addition to pack-years, we also investigated years of smoking and found this inverse association to be even stronger than that between pack-years and DNA methylation.

The main strengths of this study are the large sample size (> 1,100 women), confirmation of our 27K results in DNA from an independent group of women run on the 450K array, and verification by pyrosequencing. Limitations include few current smokers in both the 27K and the 450K data set and use of self-reporting for smoking information, which is usually reliable although sometimes underreported (Wagenknecht et al. 1992). Another possible limitation is that we have chosen to combine former smokers with current smokers when analyzing the 450K data set. There were only 12 current smokers in that data set, so estimates for current smokers would have been unstable. Previous studies (Shenker et al. 2013a, 2013b; Zeilinger et al. 2013) indicate that former smokers might be more similar to never-smokers than to current smokers, but this would only bias our results toward the null and is therefore unlikely to result in false positive associations.

## Conclusions

In the present study, we report 12 CpGs, 2 of which had not been previously described, that are differentially methylated in smokers compared with nonsmokers. Most notably, we provide the first independent replication of a cg02657160 in *CPOX,* a gene responsible for heme synthesis. We report decreased DNA methylation in smokers at this CpG, and suggest that epigenetic changes at this locus may reflect smoking-related demands for heme biosynthesis.

## Supplemental Material

(1.1 MB) PDF
