# Supplementary material for: CpG Sites Associated with Cigarette Smoking: Analysis of Epigenome-Wide Data from the Sister Study
Source: Environ Health Perspect. 2014 Apr 4;122(7):673–8. doi: 10.1289/ehp.1307480 (PMC4080519; doi:10.1289/ehp.1307480)
Supplement: (1.1 MB) PDF [file ehp.1307480.s001.pdf]

Environmental Health Perspectives doi:10.1289/ehp.1307480

Supplemental Material

CpG Sites Associated with Cigarette Smoking: Analysis of Epigenome-Wide Data from the Sister Study

Sophia Harlid, Zongli Xu, Vijayalakshmi Panduri, Dale P. Sandler, and Jack A. Taylor

Table of Contents **Page**

Smoking Variable 2

Table S1. Pyrosequencing primers. 3

Table S2. Result of regression analysis comparing methylation values of never-smokers to former or current smokers for selected CpG probes. 4

Table S3. Association between DNA methylation and smoking for the eight CpGs originally identified in the 27K dataset. 5

Table S4. Replication of the association analysis in the 612 breast cancer free (non-Case) women that were part of 27K dataset. 6

Table S5. Effects of years smoked, time since quit and interactions between the two factors on methylation changes. 7

Table S6. Effects of pack-years, time since quit and interactions between the two factors on methylation changes. 8

Table S7. Replication of previously published results. 9

Figure S1. Replication of cg02657160 (*CPOX*) using pyrosequencing. 19

References 20

Smoking Variable

The smoking classification variable ('never', 'former', and 'current smokers') is based on three raw questionnaire variables:

SM1: Have you ever smoked at least one cigarette per day for six months or longer

SM2: Have you ever smoked at least one cigarette per month for one year or longer

SM4. Have you smoked at least one cigarette per day, on average, over the past 12 months

Women who answered yes to both SM1 and SM4 were classified as current smokers and women who answered yes to SM1 and no to SM4 were classified as former smokers. Women who answered no to both SM1 and SM2 were classified as never smokers. Detailed information was collected by decade of life on smoking dose, duration, and periods of quitting for all current and former smokers, and used to construct estimates of duration, pack-years, and time since quitting.

Information on obtaining questionnaires is available through the Sister Study website: http://sisterstudy.niehs.nih.gov (NIEHS, 2014).

Table S1. Pyrosequencing primers.

Primer	Sequence
YAP1 cg15999356 Forward Primer	5’-GTATTGTATAAAGGGAGGTTTTAAAGAG-3’
YAP1 cg15999356 Reverse Primer	5’-Biotin-ACAATATCCCTTTCATAACTTATAATTCT-3’
YAP1 cg15999356 Sequencing Primer	5’-GGGAGGTTnTTAAAGAGT-3’
CPOX cg02657160 Forward Primer	5’-GGTTTAGTGGTTTTTGTTTGAAAATATAAG-3’
CPOX cg02657160 Reverse Primer	5’-Biotin-AACCCCACATCTTAAATAAAAACTT-3’
CPOX cg02657160 Sequencing Primer	5’-GTTAGTATTATAGTTTAAAATTTGT-3’

Table S2. Result of regression analysis comparing methylation values of never-smokers to former or current smokers for selected CpG probes.

CpG	Symbol	Former smoker: Coefficient	Former smoker: P	Current smoker: Coefficient	Current smoker: P
cg03636183	F2RL3	-1.723E-02	2.255E-18	-7.021E-02	3.833E-67
cg19859270	GPR15	-6.769E-03	5.622E-14	-2.631E-02	1.245E-51
cg09837977	LRRN3	-7.818E-03	5.672E-09	-1.668E-02	2.966E-11
cg26764244	GNG12	-1.020E-02	1.725E-06	-2.105E-02	1.013E-07
cg16254309	CNTNAP2	-3.161E-03	5.173E-04	-1.017E-02	2.519E-09
cg13500388	CBFB	-5.248E-03	6.547E-03	-1.925E-02	8.435E-08
cg11314684	AKT3	-4.128E-03	3.540E-02	-2.000E-02	4.530E-08
cg23667432	ALPP	-2.187E-03	1.293E-01	-1.412E-02	1.484E-07
cg13668129	HNRPUL1	-1.617E-03	1.323E-01	-1.017E-02	3.879E-07
cg18660898	MLLT11	-2.410E-04	7.731E-01	-7.471E-03	1.393E-06
cg25426302	PRRT1	-9.345E-04	4.318E-01	-1.088E-02	1.128E-06
cg15344028	ICOS	-2.382E-03	3.878E-01	-2.481E-02	1.187E-06
cg22335340	PTPN6	-2.949E-03	4.370E-02	-1.220E-02	8.549E-06
cg10084993	SLC9A3R2	-5.401E-03	1.142E-02	-1.684E-02	1.872E-05
cg22471346	GAS7	-2.944E-04	3.037E-01	2.177E-03	3.865E-05
cg21474838	KLRD1	5.509E-03	2.597E-04	9.583E-03	5.464E-04
cg25809905	ITGA2B	2.515E-03	4.982E-01	3.124E-02	6.153E-06
cg13185177	GP5	8.017E-03	5.762E-03	2.201E-02	3.949E-05

Table S3. Association between DNA methylation and smoking for the eight CpGs originally identified in the 27K dataset. Results are presented both with and without adjustments, as well as the association with passive smoking.

CpG	Gene	Active smoking: P	Active smoking: FDR	Active smoking: P[a]	Active smoking: FDR[a]	Association with passive smoking
cg03636183	*F2RL3*	9.28E-70	1.89E-65	4.70E-59	9.74E-55	6.99E-01
cg19859270	*GPR15*	2.93E-53	2.99E-49	1.37E-41	1.42E-37	9.57E-01
cg09837977	*LRRN3*	1.71E-14	1.16E-10	1.61E-11	1.11E-07	6.68E-01
cg26764244	*GNG12*	9.03E-10	4.60E-06	3.19E-07	1.65E-03	7.63E-01
cg16254309	*CNTNAP2*	1.88E-09	7.69E-06	1.44E-06	4.97E-03	5.11E-01
cg13500388	*CBFB*	1.77E-07	6.00E-04	1.56E-05	2.94E-02	5.69E-01
cg11314684	*AKT3*	2.14E-07	6.23E-04	1.13E-06	4.67E-03	5.09E-01
cg22335340	*PTPN6*	2.86E-05	3.99E-02	1.73E-04	1.80E-01	9.47E-01

[a]Adjusted for passive smoking, maternal smoking, parity, and menopause status.

Table S4. Replication of the association analysis in the 612 breast cancer free (non-Case) women that were part of 27K dataset. When we repeated the analysis in non-Case women six of the eight probes from the primary analysis remained significant under the same criteria. Two probes failed to reach significance but these two (cg13500388 in CBFB and cg11314684 in AKT3) have been independently identified by Sun et al. 2013 making it likely that the negative results are caused by the small sample size.

CpG	Gene	Cases+nonCases (n=908):P	Cases+nonCases (n=908):FDR	nonCases only (n=612):P	nonCases only (n=612):FDR
cg03636183	*F2RL3*	9.3E-70	1.9E-65	4.5E-53	1.2E-48
cg19859270	*GPR15*	2.9E-53	3.0E-49	2.6E-33	3.4E-29
cg09837977	*LRRN3*	1.7E-14	1.2E-10	1.6E-08	1.4E-04
cg26764244	*GNG12*	9.0E-10	4.6E-06	8.1E-08	3.5E-04
cg16254309	*CNTNAP2*	1.9E-09	7.7E-06	7.9E-08	3.5E-04
cg13500388	*CBFB*	1.8E-07	6.0E-04	6.7E-04	2.6E-01
cg11314684	*AKT3*	2.1E-07	6.2E-04	5.2E-04	2.6E-01
cg22335340	*PTPN6*	2.9E-05	4.0E-02	2.7E-08	1.7E-04

Table S5. Effects of years smoked, time since quit and interactions between the two factors on methylation changes. Statistical tests were done separately in current smokers and former smokers using a multiple linear regression model adjusting for age at blood draw.

CpG	Gene	Current smoker, Years smoked: Coef.	Current smoker, Years smoked: P	Former smoker, Years smoked: Coef.	Former smoker, Years smoked: P	Former smoker, Time since quit: Coef.	Former smoker, Time since quit: P	Interaction P value
27K dataset								
cg03636183	*F2RL3*	-2.77E-03	1.71E-02*	-1.61E-03	2.54E-17*	1.49E-03	9.87E-16*	2.73E-04*
cg19859270	*GPR15*	-1.01E-03	1.82E-02*	-6.19E-04	1.89E-16*	5.34E-04	6.31E-13*	1.98E-02*
cg09837977	*LRRN3*	-8.47E-04	3.45E-01	-8.20E-04	1.70E-05*	5.96E-04	1.42E-03*	8.76E-01
cg26764244	*GNG12*	-1.14E-03	1.12E-01	-3.57E-04	3.74E-02*	2.33E-04	1.63E-01	1.24E-01
cg16254309	*CNTNAP2*	4.52E-04	2.77E-01	-3.55E-04	3.37E-04*	2.56E-04	8.18E-03*	8.20E-01
cg13500388	*CBFB*	4.57E-04	5.12E-01	-5.52E-04	2.80E-03*	4.03E-04	2.53E-02*	3.12E-01
cg11314684	*AKT3*	-6.84E-05	9.19E-01	-6.53E-04	3.82E-04*	6.59E-04	2.32E-04*	6.48E-01
cg22335340	*PTPN6*	-6.07E-04	1.99E-01	-1.68E-04	2.90E-01	2.02E-04	1.93E-01	2.91E-03*
450K dataset								
cg02657160	*CPOX*	-7.28E-04	2.18E-01	-1.19E-04	5.15E-01	2.51E-04	1.86E-01	5.32E-01
cg15999356	*YAP1*	-1.96E-03	5.23E-01	8.53E-04	2.74E-01	-1.17E-04	8.87E-01	7.87E-01
cg05575921	*AHRR*	-5.19E-03	2.66E-01	-2.58E-03	3.25E-06*	2.20E-03	2.47E-04*	6.62E-02
cg06644428	*2q37.1*	-4.50E-04	4.48E-01	-1.38E-03	1.44E-03*	1.44E-03	1.43E-03*	4.41E-01
cg05951221	*2q37.1*	-3.49E-03	2.06E-01	-3.10E-03	6.69E-05*	3.22E-03	7.38E-05*	7.39E-01
Pyrosequencing								
cg02657160	*CPOX*	-6.12E-02	1.14E-01	-5.00E-02	2.43E-03*	5.45E-02	4.31E-04*	9.87E-01
cg15999356	*YAP1*	2.10E-01	1.88E-01	-7.90E-02	2.43E-01	9.06E-02	1.60E-01	2.49E-01

*p<0.05.

Table S6. Effects of pack-years, time since quit and interactions between the two factors on methylation changes. Statistical tests were done separately in current smokers and former smokers using a multiple linear regression model adjusting for age at blood draw.

CpG	Gene	Current smoker, Pack-year: Coef.	Current smoker, Pack-year: P	Former smoker, Pack-year: Coef.	Former smoker, Pack-year: P	Former smoker, Time since quit: Coef.	Former smoker, Time since quit: P	Interaction P value
27K dataset								
cg03636183	*F2RL3*	-6.63E-04	3.14E-01	-9.07E-04	3.04E-10*	1.49E-03	9.87E-16*	2.56E-04*
cg19859270	*GPR15*	-2.07E-04	3.38E-01	-4.10E-04	3.77E-13*	5.34E-04	6.31E-13*	6.56E-02
cg09837977	*LRRN3*	-3.70E-04	4.24E-01	-5.06E-04	4.10E-04*	5.96E-04	1.42E-03*	8.54E-01
cg26764244	*GNG12*	-1.09E-04	7.70E-01	-2.45E-04	5.48E-02	2.33E-04	1.63E-01	3.94E-01
cg16254309	*CNTNAP2*	1.24E-04	5.57E-01	-2.41E-04	9.61E-04*	2.56E-04	8.18E-03*	8.35E-01
cg13500388	*CBFB*	1.79E-04	6.04E-01	-3.30E-04	1.58E-02*	4.03E-04	2.53E-02*	4.09E-01
cg11314684	*AKT3*	-1.17E-04	7.36E-01	-2.70E-04	4.83E-02*	6.59E-04	2.32E-04*	8.08E-01
cg22335340	*PTPN6*	-2.76E-04	2.65E-01	-1.46E-04	2.13E-01	2.02E-04	1.93E-01	6.86E-02
450K dataset								
cg02657160	*CPOX*	-3.67E-04	1.47E-01	-1.94E-04	1.91E-01	2.51E-04	1.86E-01	5.25E-01
cg15999356	*YAP1*	-1.24E-03	3.42E-01	6.19E-04	3.28E-01	-1.17E-04	8.87E-01	4.04E-01
cg05575921	*AHRR*	-2.81E-03	4.47E-01	-2.18E-03	7.36E-07*	2.20E-03	2.47E-04*	3.67E-02*
cg06644428	*2q37.1*	1.36E-04	5.98E-01	-8.92E-04	1.15E-02*	1.44E-03	1.43E-03*	3.94E-01
cg05951221	*2q37.1*	-1.86E-04	8.76E-01	-2.54E-03	5.93E-05*	3.22E-03	7.38E-05*	4.17E-01
Pyrosequencing								
cg02657160	*CPOX*	5.17E-03	7.98E-01	-3.64E-02	1.64E-04*	5.45E-02	4.31E-04*	9.26E-01
cg15999356	*YAP1*	8.89E-02	2.68E-01	-9.83E-02	1.52E-02	9.06E-02	1.60E-01	2.83E-01

*p<0.05.

Table S7. Replication of previously published results. All CpGs in this list have been reported to be associated with smoking status in at least one study. CpGs are listed based on number of studies that have reported an association for that CpG.

CpG	CHR	Gene	27K: P	450K: P	Source[a]
cg19859270	3	*GPR15*	2.93E-53	3.78E-04	(Breitling et al. 2011; Sun et al. 2013; Wan et al. 2012; Zeilinger et al. 2013)
cg03636183	19	*F2RL3*	9.28E-70	1.97E-07	(Breitling et al. 2011; Sun et al. 2013; Wan et al. 2012; Zeilinger et al. 2013)
cg06644428	2	*2q37*	NA[b]	2.56E-07	(Sun et al. 2013; Zeilinger et al. 2013; Shenker et al. 2013)
cg05951221	2	*2q37*	NA[b]	7.06E-07	(Sun et al. 2013; Zeilinger et al. 2013; Shenker et al. 2013)
cg21566642	2	*2q37*	NA[b]	2.98E-06	(Sun et al. 2013; Zeilinger et al. 2013; Shenker et al. 2013)
cg01940273	2	*2q37*	NA[b]	8.06E-04	(Sun et al. 2013; Zeilinger et al. 2013; Shenker et al. 2013)
cg23576855	5	*AHRR*	NA[b]	0.02	(Sun et al. 2013; Zeilinger et al. 2013; Shenker et al. 2013)
cg05575921	5	*AHRR*	NA[b]	2.60E-07	(Sun et al. 2013; Zeilinger et al. 2013; Shenker et al. 2013)
cg21161138	5	*AHRR*	NA[b]	4.55E-03	(Sun et al. 2013; Zeilinger et al. 2013; Shenker et al. 2013)
cg06126421	6	*6p21*	NA[b]	6.70E-06	(Sun et al. 2013; Zeilinger et al. 2013; Shenker et al. 2013)
cg15258980	2	*ARHGAP25*	0.09	0.75	(Sun et al. 2013; Wan et al. 2012)
cg05445326	3	*TM4SF19*	0.4	0.49	(Sun et al. 2013; Wan et al. 2012)
cg16254309	7	*CNTNAP2*	1.88E-09	0.01	(Sun et al. 2013; Wan et al. 2012)
cg09837977	7	*LRRN3*	1.71E-14	5.02E-04	(Sun et al. 2013; Wan et al. 2012)
cg09084200	11	*NCAPD3*	6.97E-03	0.03	(Sun et al. 2013; Wan et al. 2012)

CpG	CHR	Gene	27K: P	450K: P	Source[a]
cg15691199	14	*CEBPE*	1.20E-04	0.03	(Sun et al. 2013; Wan et al. 2012)
cg21917349	15	*APBA2*	0.11	0.89	(Sun et al. 2013; Wan et al. 2012)
cg01500140	19	*LIM2*	5.26E-04	0.01	(Sun et al. 2013; Wan et al. 2012)
cg10161121	1	*FASLG*	0.84	0.74	(Wan et al. 2012)
cg18881723	1	*SLAMF1*	0.04	0.23	(Wan et al. 2012)
cg13247990	3	*MYLK*	0.41	0.34	(Wan et al. 2012)
cg24262469	3	*TIPARP*	0.03	0.81	(Wan et al. 2012)
cg02564523	7	*ORA12*	2.95E-03	0.29	(Wan et al. 2012)
cg01988129	8	*ADHFE1*	5.41E-03	0.42	(Wan et al. 2012)
cg12044210	15	*APBA2*	NA[b]	0.45	(Wan et al. 2012)
cg09155905	17	*FNDC8*	0.08	0.88	(Wan et al. 2012)
cg11314684	1	*AKT3*	2.14E-07	9.31E-04	(Sun et al. 2013)
cg04983977	1	*GPR25*	0.24	0.57	(Sun et al. 2013)
cg17791651	1	*POU3F1*	NA[b]	0.25	(Sun et al. 2013)
cg03330058	3	*ABTB1*	0.18	0.49	(Sun et al. 2013)
cg13745870	3	*SPATA12*	NA[b]	0.81	(Sun et al. 2013)
cg03340878	6	*OR2B6*	0.000165	0.81	(Sun et al. 2013)
cg14223444	9	*NCBP1*	0.05	0.59	(Sun et al. 2013)
cg13633560	11	*LRRC32*	0.02	0.07	(Sun et al. 2013)
cg00353953	12	*ZNF384*	0.01	0.47	(Sun et al. 2013)
cg26259865	16	*LOC124220*	0.02	0.09	(Sun et al. 2013)
cg13668129	19	*HNRPUL1*	2.36E-06	0.3	(Sun et al. 2013)
cg12547807	1	NA[c]	NA[b]	5.15E-03	(Zeilinger et al. 2013)

CpG	CHR	Gene	27K: P	450K: P	Source[a]
cg04885881	1	NA[c]	NA[b]	0.04	(Zeilinger et al. 2013)
cg21393163	1	NA[c]	NA[b]	4.62E-03	(Zeilinger et al. 2013)
cg27537125	1	NA[c]	NA[b]	6.57E-03	(Zeilinger et al. 2013)
cg23090529	1	NA[c]	NA[b]	0.81	(Zeilinger et al. 2013)
cg21140898	1	NA[c]	NA[b]	0.4	(Zeilinger et al. 2013)
cg03547355	1	NA[c]	NA[b]	8.77E-04	(Zeilinger et al. 2013)
cg17819085	1	NA[c]	NA[b]	5.04E-03	(Zeilinger et al. 2013)
cg08709672	1	*AVPR1B*	NA[b]	0.01	(Zeilinger et al. 2013)
cg20295214	1	*AVPR1B*	NA[b]	3.65E-03	(Zeilinger et al. 2013)
cg19713429	1	*CAPZB*	NA[b]	0.16	(Zeilinger et al. 2013)
cg09662411	1	*GFI1*	NA[b]	0.1	(Zeilinger et al. 2013)
cg18146737	1	*GFI1*	NA[b]	0.02	(Zeilinger et al. 2013)
cg12876356	1	*GFI1*	NA[b]	0.01	(Zeilinger et al. 2013)
cg18316974	1	*GFI1*	NA[b]	0.01	(Zeilinger et al. 2013)
cg09935388	1	*GFI1*	NA[b]	0.01	(Zeilinger et al. 2013)
cg25189904	1	*GNG12*	NA[b]	2.94E-06	(Zeilinger et al. 2013)
cg15542713	1	*HIVEP3*	NA[b]	0.02	(Zeilinger et al. 2013)
cg24049493	1	*HIVEP3*	NA[b]	5.20E-04	(Zeilinger et al. 2013)
cg11231349	1	*NOS1AP*	NA[b]	0.13	(Zeilinger et al. 2013)
cg19406367	1	*SGIP1*	NA[b]	0.09	(Zeilinger et al. 2013)
cg09469355	1	*SKI*	NA[b]	8.77E-03	(Zeilinger et al. 2013)
cg08884752	1	*SKI*	NA[b]	0.02	(Zeilinger et al. 2013)
cg21913886	1	*TMEM51*	NA[b]	0.99	(Zeilinger et al. 2013)

CpG	CHR	Gene	27K: P	450K: P	Source[a]
cg23079012	2	NA[c]	NA[b]	0.12	(Zeilinger et al. 2013)
cg27241845	2	NA[c]	NA[b]	2.61E-03	(Zeilinger et al. 2013)
cg03329539	2	NA[c]	NA[b]	1.03E-03	(Zeilinger et al. 2013)
cg13193840	2	NA[c]	NA[b]	0.02	(Zeilinger et al. 2013)
cg23667432	2	*ALPP*	9.46E-07	0.22	(Zeilinger et al. 2013)
cg03188382	2	*ALPP*	NA[b]	0.18	(Zeilinger et al. 2013)
cg19713851	2	*ALPP*	NA[b]	0.06	(Zeilinger et al. 2013)
cg06635952	2	*ANXA4*	NA[b]	0.19	(Zeilinger et al. 2013)
cg26271591	2	*NFE2L2*	NA[b]	0.25	(Zeilinger et al. 2013)
cg15693572	3	NA[c]	NA[b]	7.90E-03	(Zeilinger et al. 2013)
cg23480021	3	NA[c]	NA[b]	1.51E-03	(Zeilinger et al. 2013)
cg03274391	3	NA[c]	NA[b]	7.49E-04	(Zeilinger et al. 2013)
cg15417641	3	*CACNA1D*	NA[b]	0.00118	(Zeilinger et al. 2013)
cg00336149	3	*CACNA1D*	NA[b]	6.37E-03	(Zeilinger et al. 2013)
cg21188533	3	*CACNA1D*	NA[b]	2.25E-03	(Zeilinger et al. 2013)
cg25197194	3	*CCDC48*	NA[b]	0.02	(Zeilinger et al. 2013)
cg02657160	3	*CPOX*	NA[b]	7.28E-07	(Zeilinger et al. 2013)
cg00501876	3	*CSRNP1*	NA[b]	0.05	(Zeilinger et al. 2013)
cg18642234	3	*GPX1*	NA[b]	0.18	(Zeilinger et al. 2013)
cg08202836	3	*LRRC33*	NA[b]	5.53E-01	(Zeilinger et al. 2013)
cg17024919	3	*ZNF385D*	NA[b]	0.07	(Zeilinger et al. 2013)
cg19719391	4	NA[c]	NA[b]	0.89	(Zeilinger et al. 2013)
cg24556382	4	*GALNT7*	NA[b]	0.21	(Zeilinger et al. 2013)

CpG	CHR	Gene	27K: P	450K: P	Source[a]
cg21121843	4	*HTT*	NA[b]	0.74	(Zeilinger et al. 2013)
cg12513616	5	NA[c]	NA[b]	1.39E-03	(Zeilinger et al. 2013)
cg26703534	5	*AHRR*	NA[b]	0.13	(Zeilinger et al. 2013)
cg25648203	5	*AHRR*	NA[b]	0.05	(Zeilinger et al. 2013)
cg24090911	5	*AHRR*	NA[b]	0.03	(Zeilinger et al. 2013)
cg23916896	5	*AHRR*	NA[b]	0.02	(Zeilinger et al. 2013)
cg17924476	5	*AHRR*	NA[b]	0.04	(Zeilinger et al. 2013)
cg14817490	5	*AHRR*	NA[b]	0.62	(Zeilinger et al. 2013)
cg12806681	5	*AHRR*	NA[b]	7.20E-03	(Zeilinger et al. 2013)
cg11902777	5	*AHRR*	NA[b]	0.01	(Zeilinger et al. 2013)
cg11554391	5	*AHRR*	NA[b]	2.77E-04	(Zeilinger et al. 2013)
cg08606254	5	*AHRR*	NA[b]	0.03	(Zeilinger et al. 2013)
cg03991871	5	*AHRR*	NA[b]	5.35E-05	(Zeilinger et al. 2013)
cg03604011	5	*AHRR*	NA[b]	0.04	(Zeilinger et al. 2013)
cg01899089	5	*AHRR*	NA[b]	2.80E-03	(Zeilinger et al. 2013)
cg01097768	5	*AHRR*	NA[b]	0.03	(Zeilinger et al. 2013)
cg14580211	5	*C5orf62*	NA[b]	7.49E-04	(Zeilinger et al. 2013)
cg13039251	5	*PDZD2*	NA[b]	0.29	(Zeilinger et al. 2013)
cg05673882	5	*POLK*	NA[b]	3.46E-03	(Zeilinger et al. 2013)
cg16786458	5	*PPARGC1B*	2.52E-03	0.21	(Zeilinger et al. 2013)
cg26908328	5	*SERINC5*	NA[b]	0.72	(Zeilinger et al. 2013)
cg24859433	6	NA[c]	NA[b]	0.000061	(Zeilinger et al. 2013)
cg15342087	6	NA[c]	NA[b]	0.09	(Zeilinger et al. 2013)

CpG	CHR	Gene	27K: P	450K: P	Source[a]
cg14753356	6	NA[c]	NA[b]	0.03	(Zeilinger et al. 2013)
cg01882991	6	NA[c]	NA[b]	0.02	(Zeilinger et al. 2013)
cg15474579	6	*CDKN1A*	NA[b]	0.06	(Zeilinger et al. 2013)
cg00931843	6	*TIAM2*	NA[b]	0.04	(Zeilinger et al. 2013)
cg17619755	6	*VARS*	NA[b]	3.27E-03	(Zeilinger et al. 2013)
cg10807309	6	*VARS*	NA[b]	0.38	(Zeilinger et al. 2013)
cg03440944	7	*C7orf40*	NA[b]	0.13	(Zeilinger et al. 2013)
cg08972170	7	*C7orf41*	NA[b]	0.2	(Zeilinger et al. 2013)
cg25949550	7	*CNTNAP2*	NA[b]	1.12E-03	(Zeilinger et al. 2013)
cg21322436	7	*CNTNAP2*	NA[b]	2.26E-03	(Zeilinger et al. 2013)
cg17372101	7	*CNTNAP2*	NA[b]	0.03	(Zeilinger et al. 2013)
cg11207515	7	*CNTNAP2*	NA[b]	0.08	(Zeilinger et al. 2013)
cg19717773	7	*GNA12*	NA[b]	0.32	(Zeilinger et al. 2013)
cg00921574	7	*INTS1*	NA[b]	0.69	(Zeilinger et al. 2013)
cg02451831	7	*KIAA0087*	NA[b]	0.19	(Zeilinger et al. 2013)
cg22132788	7	*MYO1G*	NA[b]	0.01	(Zeilinger et al. 2013)
cg19089201	7	*MYO1G*	NA[b]	0.01	(Zeilinger et al. 2013)
cg12803068	7	*MYO1G*	NA[b]	1.02E-03	(Zeilinger et al. 2013)
cg07826859	7	*MYO1G*	NA[b]	0.13	(Zeilinger et al. 2013)
cg04180046	7	*MYO1G*	NA[b]	0.02	(Zeilinger et al. 2013)
cg25305703	8	NA[c]	NA[b]	0.21	(Zeilinger et al. 2013)
cg24540678	8	NA[c]	NA[b]	0.02	(Zeilinger et al. 2013)
cg19589396	8	NA[c]	NA[b]	7.72E-03	(Zeilinger et al. 2013)

CpG	CHR	Gene	27K: P	450K: P	Source[a]
cg13518625	8	NA[c]	NA[b]	0.25	(Zeilinger et al. 2013)
cg12075928	8	*PTK2*	NA[b]	8.13E-03	(Zeilinger et al. 2013)
cg12276019	8	*XKR6*	NA[b]	0.49	(Zeilinger et al. 2013)
cg26361535	8	*ZC3H3*	NA[b]	0.04	(Zeilinger et al. 2013)
cg13787850	9	NA[c]	NA[b]	0.2	(Zeilinger et al. 2013)
cg01692968	9	NA[c]	NA[b]	1.61E-05	(Zeilinger et al. 2013)
cg13910681	9	*FAM102A*	NA[b]	0.83	(Zeilinger et al. 2013)
cg22539182	10	*NA*[c]	NA[b]	0.1	(Zeilinger et al. 2013)
cg25953130	10	*ARID5B*	NA[b]	0.13	(Zeilinger et al. 2013)
cg27312979	10	*SORBS1*	NA[b]	0.3	(Zeilinger et al. 2013)
cg25421530	10	*SORBS1*	NA[b]	0.73	(Zeilinger et al. 2013)
cg16611234	11	NA[c]	NA[b]	0.2	(Zeilinger et al. 2013)
cg03234777	11	*AMICA1*	NA[b]	0.14	(Zeilinger et al. 2013)
cg01901332	11	*ARRB1*	NA[b]	0.02	(Zeilinger et al. 2013)
cg19254163	11	*GPR44*	NA[b]	0.07	(Zeilinger et al. 2013)
cg26963277	11	*KCNQ1OT1*	NA[b]	0.08	(Zeilinger et al. 2013)
cg16556677	11	*KCNQ1OT1*	NA[b]	0.78	(Zeilinger et al. 2013)
cg07123182	11	*KCNQ1OT1*	NA[b]	0.19	(Zeilinger et al. 2013)
cg01744331	11	*KCNQ1OT1*	NA[b]	0.15	(Zeilinger et al. 2013)
cg21611682	11	*LRP5*	NA[b]	6.10E-04	(Zeilinger et al. 2013)
cg14624207	11	*LRP5*	NA[b]	0.18	(Zeilinger et al. 2013)
cg04039799	11	*NAV2*	NA[b]	0.13	(Zeilinger et al. 2013)
cg23771366	11	*PRSS23*	NA[b]	1.16E-03	(Zeilinger et al. 2013)

CpG	CHR	Gene	27K: P	450K: P	Source[a]
cg11660018	11	*PRSS23*	NA[b]	0.01	(Zeilinger et al. 2013)
cg09197783	11	*SLC43A3*	NA[b]	0.1	(Zeilinger et al. 2013)
cg02583484	12	*HNRNPA1*	NA[b]	0.48	(Zeilinger et al. 2013)
cg04158018	12	*NFE2*	NA[b]	0.32	(Zeilinger et al. 2013)
cg26282236	12	*RAD52*	NA[b]	0.63	(Zeilinger et al. 2013)
cg23681440	13	NA[c]	NA[b]	0.31	(Zeilinger et al. 2013)
cg06885459	13	*MCF2L*	NA[b]	0.13	(Zeilinger et al. 2013)
cg25491122	13	*PCDH9*	NA[b]	0.45	(Zeilinger et al. 2013)
cg23126342	13	*PCDH9*	NA[b]	0.53	(Zeilinger et al. 2013)
cg17487894	13	*RASA3*	NA[b]	0.26	(Zeilinger et al. 2013)
cg13038618	14	NA[c]	NA[b]	0.13	(Zeilinger et al. 2013)
cg01208318	14	NA[c]	NA[b]	0.33	(Zeilinger et al. 2013)
cg24996979	14	*C14orf43*	NA[b]	0.19	(Zeilinger et al. 2013)
cg22851561	14	*C14orf43*	NA[b]	9.52E-03	(Zeilinger et al. 2013)
cg13976502	14	*C14orf43*	NA[b]	0.08	(Zeilinger et al. 2013)
cg10919522	14	*C14orf43*	NA[b]	0.13	(Zeilinger et al. 2013)
cg01731783	14	*C14orf43*	NA[b]	0.1	(Zeilinger et al. 2013)
cg05875421	14	*GPR68*	NA[b]	0.3	(Zeilinger et al. 2013)
cg11730703	14	*INF2*	NA[b]	0.56	(Zeilinger et al. 2013)
cg05284742	14	*ITPK1*	NA[b]	0.09	(Zeilinger et al. 2013)
cg06819357	14	*TECPR2*	NA[b]	0.09	(Zeilinger et al. 2013)
cg26242531	14	*ZFYVE21*	NA[b]	0.02	(Zeilinger et al. 2013)
cg05194346	15	NA[c]	NA[b]	0.24	(Zeilinger et al. 2013)

CpG	CHR	Gene	27K: P	450K: P	Source[a]
cg23161492	15	*ANPEP*	NA[b]	2.71E-03	(Zeilinger et al. 2013)
cg11152412	15	*EDC3*	NA[b]	0.25	(Zeilinger et al. 2013)
cg03489965	15	*LOC390594*	NA[b]	0.07	(Zeilinger et al. 2013)
cg18335991	15	*SEMA7A*	NA[b]	0.05	(Zeilinger et al. 2013)
cg00310412	15	*SEMA7A*	NA[b]	0.26	(Zeilinger et al. 2013)
cg15022400	15	*TRIM69*	NA[b]	0.83	(Zeilinger et al. 2013)
cg03155159	16	NA[c]	NA[b]	0.45	(Zeilinger et al. 2013)
cg01207684	16	*ADCY9*	NA[b]	0.04	(Zeilinger et al. 2013)
cg09099830	16	*ITGAL*	NA[b]	0.09	(Zeilinger et al. 2013)
cg02186444	17	*ARMC7*	NA[b]	0.74	(Zeilinger et al. 2013)
cg16255816	17	*HAP1*	NA[b]	0.06	(Zeilinger et al. 2013)
cg03373393	17	*HAP1*	NA[b]	0.61	(Zeilinger et al. 2013)
cg23621097	17	*HIC1*	NA[b]	0.37	(Zeilinger et al. 2013)
cg00911794	17	*HIC1*	NA[b]	0.24	(Zeilinger et al. 2013)
cg25809905	17	*ITGA2B*	3.42E-05	0.77	(Zeilinger et al. 2013)
cg07251887	17	*LOC100130933*	NA[b]	0.02	(Zeilinger et al. 2013)
cg21280392	17	*PHOSPHO1*	NA[b]	0.32	(Zeilinger et al. 2013)
cg19572487	17	*RARA*	NA[b]	5.95E-04	(Zeilinger et al. 2013)
cg09858022	17	*RARA*	NA[b]	0.02	(Zeilinger et al. 2013)
cg04956244	17	*RARA*	NA[b]	0.05	(Zeilinger et al. 2013)
cg07465627	17	*STXBP4*	NA[b]	0.58	(Zeilinger et al. 2013)
cg06459104	18	*EPB41L3*	NA[b]	0.11	(Zeilinger et al. 2013)
cg00073090	19	NA[c]	NA[b]	0.26	(Zeilinger et al. 2013)

CpG	CHR	Gene	27K: P	450K: P	Source[a]
cg15159987	19	*CPAMD8*	NA[b]	0.09	(Zeilinger et al. 2013)
cg23973524	19	*CRTC1*	NA[b]	0.03	(Zeilinger et al. 2013)
cg22649124	19	*LGALS7B*	NA[b]	0.75	(Zeilinger et al. 2013)
cg00835193	19	*LINGO3*	NA[b]	0.48	(Zeilinger et al. 2013)
cg11902728	19	*MAG*	NA[b]	0.23	(Zeilinger et al. 2013)
cg15187398	19	*MOBKL2A*	NA[b]	0.28	(Zeilinger et al. 2013)
cg07381806	19	*MOBKL2A*	NA[b]	0.02	(Zeilinger et al. 2013)
cg11701312	19	*RPS5*	NA[b]	0.4	(Zeilinger et al. 2013)
cg16201146	20	NA[c]	NA[b]	0.42	(Zeilinger et al. 2013)
cg07339236	20	*ATP9A*	NA[b]	0.02	(Zeilinger et al. 2013)
cg22635096	21	*ADARB1*	NA[b]	0.06	(Zeilinger et al. 2013)
cg23110422	21	*ETS2*	NA[b]	0.02	(Zeilinger et al. 2013)
cg00871610	21	*MIR802*	NA[b]	7.96E-03	(Zeilinger et al. 2013)
cg06595162	21	*NCRNA00114*	NA[b]	0.28	(Zeilinger et al. 2013)
cg01127300	22	NA[c]	NA[b]	0.06	(Zeilinger et al. 2013)
cg02532700	22	*NCF4*	NA[b]	0.95	(Zeilinger et al. 2013)

[a]Refers to previous publications where the CpG has been reported. [b]Not included on the 27K array. [c]No Known gene.

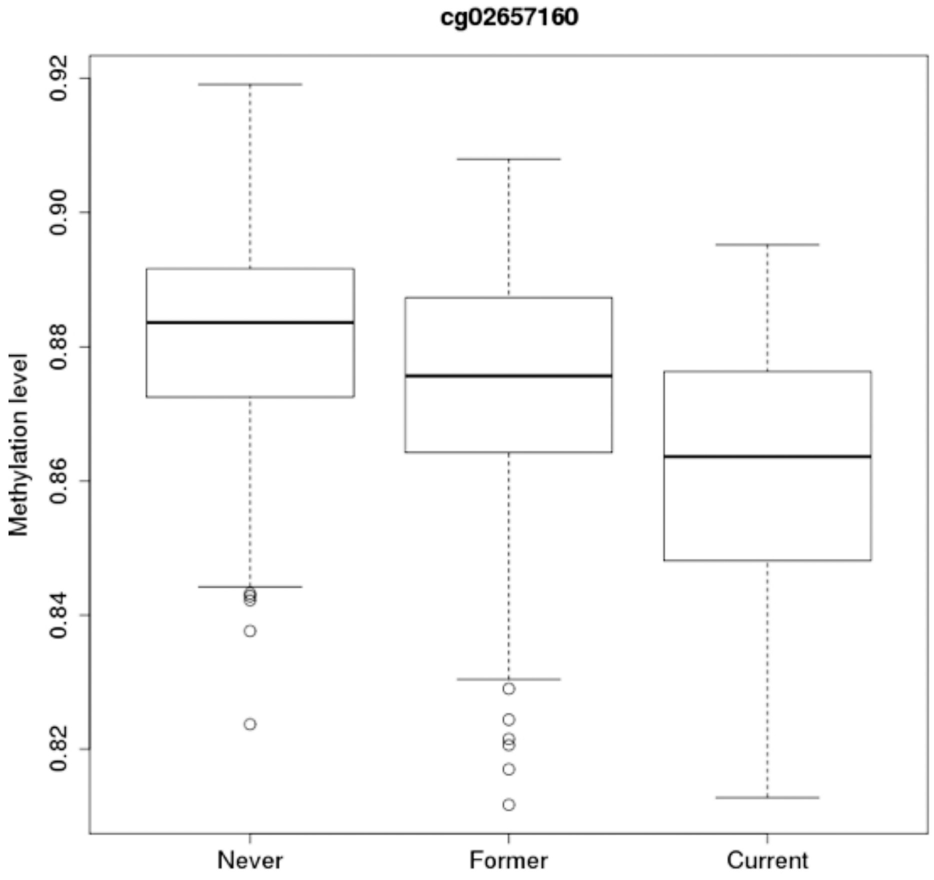

Figure S1. Replication of cg02657160 (*CPOX*) using pyrosequencing. A two-way ANOVA test comparing never / former smokers ($P=7.56\times10^{-4}$) and never / current smokers ($P=2.77\times10^{-14}$) showed significantly decreased methylation in the groups exposed to smoking. Shown are the highest methylation datum within 1.5 inter-quantile-range (IQR) of the upper quartile, upper quantile, median, lower quartile, and the lowest methylation datum within 1.5 IQR of the lower quartile. Methylation data outside of 1.5 IQR from lower or upper quartile are plotted as open circles.

References

Breitling LP, Yang R, Korn B, Burwinkel B, Brenner H. 2011. Tobacco-smoking-related differential DNA methylation: 27K discovery and replication. Am J Hum Genet 88: 450-457.

NIEHS (National Institute of Environmental Health Sciences) 2014. The Sister Study Cohort Description. Available: http://sisterstudy.niehs.nih.gov [accessed 19 March 2014].

Shenker NS, Polidoro S, van Veldhoven K, Sacerdote C, Ricceri F, Birrell MA, et al. 2013. Epigenome-wide association study in the european prospective investigation into cancer and nutrition (EPIC-Turin) identifies novel genetic loci associated with smoking. Hum Mol Genet 22:843-851.

Sun Y, Smith A, Conneely K, Chang Q, Li W, Lazarus A, et al. 2013. Epigenomic association analysis identifies smoking-related DNA methylation sites in African Americans. Hum Genet 132:1027-1037

Wan ES, Qiu W, Baccarelli A, Carey VJ, Bacherman H, Rennard SI, et al. 2012. Cigarette smoking behaviors and time since quitting are associated with differential DNA methylation across the human genome. Hum Mol Genet 21: 3073-3082.

Zeilinger S, Kühnel B, Klopp N, Baurecht H, Kleinschmidt A, Gieger C, et al. 2013. Tobacco smoking leads to extensive genome-wide changes in DNA methylation. PloS one 8:1-14.
